# Checkpoint kinase inhibitor AZD7762 strongly sensitises urothelial carcinoma cells to gemcitabine

**DOI:** 10.1186/s13046-016-0473-1

**Published:** 2017-01-03

**Authors:** Makoto Isono, Michèle J. Hoffmann, Maria Pinkerneil, Akinori Sato, Martin Michaelis, Jindrich Cinatl, Günter Niegisch, Wolfgang A. Schulz

**Affiliations:** 1 Department of Urology, Medical Faculty, Heinrich-Heine-University Düsseldorf, Universitätsstraße 1, 40225 Düsseldorf, Germany; 2 Department of Urology, National Defense Medical College, Namiki 3-2, 359-8513 Tokorozawa, Japan; 3 Centre for Molecular Processing and School of Biosciences, University of Kent, Canterbury, CT2 7NJ UK; 4 Institut für Medizinische Virologie, Klinikum der Goethe-Universität, Paul-Ehrlich‑Str. 40, 60596 Frankfurt am Main, Germany

**Keywords:** Gemcitabine, AZD7762, Checkpoint kinase, Urothelial carcinoma, Bladder cancer

## Abstract

**Background:**

More effective chemotherapies are urgently needed for bladder cancer, a major cause of morbidity and mortality worldwide. We therefore explored the efficacy of the combination of gemcitabine and AZD7762, a checkpoint kinase 1/2 (CHK1/2) inhibitor, for bladder cancer.

**Methods:**

Viability, clonogenicity, cell cycle distribution and apoptosis were assessed in urothelial cancer cell lines and various non-malignant urothelial cells treated with gemcitabine and AZD7762. DNA damage was assessed by γH2A.X and 53-BP1 staining and checkpoint activation was followed by Western blotting. Pharmacological inhibition of CHK1 and CHK2 was compared to downregulation of either CHK1 or CHK2 using siRNAs.

**Results:**

Combined use of gemcitabine and AZD7762 synergistically reduced urothelial carcinoma cell viability and colony formation relative to either single treatment. Non-malignant urothelial cells were substantially less sensitive to this drug combination. Gemcitabine plus AZD7762 inhibited cell cycle progression causing cell accumulation in S-phase. Moreover, the combination induced pronounced levels of apoptosis as indicated by an increase in the fraction of sub-G1 cells, in the levels of cleaved PARP, and in caspase 3/7 activity. Mechanistic investigations showed that AZD7762 treatment inhibited the repair of gemcitabine-induced double strand breaks by interference with CHK1, since siRNA-mediated depletion of CHK1 but not of CHK2 mimicked the effects of AZD7762.

**Conclusions:**

AZD7762 enhanced sensitivity of urothelial carcinoma cells to gemcitabine by inhibiting DNA repair and disturbing checkpoints. Combining gemcitabine with CHK1 inhibition holds promise for urothelial cancer therapy.

**Electronic supplementary material:**

The online version of this article (doi:10.1186/s13046-016-0473-1) contains supplementary material, which is available to authorized users.

## Background

Bladder cancer is a major cause of morbidity and mortality worldwide, with about 380,000 new cases and 150,000 deaths per year [1]. Unfortunately, treatment of advanced bladder cancer has progressed little over the past two decades [2]. Combination chemotherapies employing gemcitabine plus cisplatin (GC) or methotrexate plus vinblastine, doxorubicin and cisplatin (MVAC) are the first line standard regimens for advanced or metastatic bladder cancer [3]. Although the tumours often respond initially, the median overall survival (OS) of patients with advanced bladder carcinoma is only approximately 14 months, and median progression-free survival after cisplatin-based first-line therapy ranges from 7 to 9 months [4]. Furthermore, more than 50% of bladder cancer patients are unfit for cisplatin treatment because of renal dysfunction, a poor performance status, or comorbidities [5]. For patients ineligible for cisplatin the European Association of Urology (EAU) guidelines indicate chemotherapy using carboplatin and gemcitabine as a first line therapy, but the median OS is only 9.3 months [6, 7]. Therefore, more effective regimens avoiding cisplatin are urgently needed.

Genomic instability is a pervasive characteristic of many cancers, especially invasive urothelial carcinoma, the major histological subtype of bladder cancer, and DNA damage is a key factor in the evolution and treatment of cancer [8]. Upon DNA damage caused by chemotherapy, checkpoints respond to DNA damage by arresting the cell cycle to provide time for DNA repair. Checkpoint signalling following DNA damage is initiated by the proximal kinases, ataxia telangiectasia mutated (ATM) or ataxia telangiectasia and RAD3-related (ATR) [9]. ATM is recruited to and activated primarily at DNA double-strand breaks (DSBs) in conjunction with the MRE11:RAD50:NBS1 (MRN) sensor complex [10]. ATR is activated by recruitment to tracts of single strand DNA (ssDNA), and the ATR-ATRIP (ATR-interacting protein) protein kinase complex is crucial for the cellular response to replication stress and DNA damage [11]. These kinases subsequently activate the downstream serine/threonine kinases, checkpoint kinase 1 and 2 (CHK1 and CHK2). The functionally analogous kinases CHK1 and CHK2 then act as transducers in DNA-damage checkpoint signalling [12]. Upon activation, CHK1 and CHK2 phosphorylate downstream effectors such as the CDC25 family that further propagate checkpoint signalling, leading to intra-S phase and G2/M phase arrest [13]. Inhibition of checkpoint kinases abrogates DNA damage-induced cell cycle arrest allowing cells to enter mitosis despite the presence of DNA damage, which can lead to cell death. Accordingly, AZD7762, a potent CHK1/CHK2 inhibitor, possesses chemosensitizing activity in vitro and in vivo [14]. However, it remains unknown to which extent AZD7762 may promote chemotherapy-induced tumour cell death in urothelial cancer cell lines (UCCs).

Gemcitabine is a deoxycytidine analogue that has been used as a chemotherapeutic agent for more than 15 years. It disturbs DNA synthesis [15] by inhibition of deoxynucleotide biosynthetic enzymes [16], ultimately inducing apoptosis [17].

In the current study we investigated whether AZD7762 co-administration is capable of enhancing the toxicity of gemcitabine in UCCs and investigated the underlying mechanisms.

## Methods

### Cell culture

The UCCs VM-CUB1, RT-112 and T24 were obtained from the DSMZ (Braunschweig, Germany); the UM-UC-3 cell line was kindly provided by Dr. Grossman (Houston, USA). All UCCs were regularly authenticated by STR profiling (most recent fingerprint analysis shown in [18]). A human telomerase reverse transcriptase (hTERT)-immortalized normal human urothelial cell line (hTERT-NHUC) [19] was kindly provided by Dr. Knowles (Leeds, UK). The spontaneously immortalized human bladder cell line HBLAK was obtained from CELLnTEC (Bern, Switzerland). The gemcitabine resistant T24 cell line variant T24^r^GEMCI^20^ [20] was derived from the Resistant Cancer Cell Line (RCCL) collection (www.kent.ac.uk/stms/cmp/RCCL/RCCLabout.html). UCCs were routinely maintained in DMEM GlutaMAX-I (Gibco, Life Technologies, Darmstadt, Germany) supplemented with 10% foetal calf serum (GE Healthcare, Piscataway, NJ, USA) and for T24^r^GEMCI^20^ cells additionally with 20 ng/ml gemcitabine (Selleck Chemicals, Munich, Germany). hTERT-NHUC was cultured in keratinocyte serum-free medium (Gibco, Life Technologies, Darmstadt, Germany) supplemented with 0.25 ng/mL EGF, 12.5 μg/mL bovine pituitary extract and 1:100 insulin-transferrin-selenium (Gibco, Life Technologies), 0.35 μg/mL N-epinephrine, and 0.33 mg/mL hydrocortisone (Sigma Aldrich, Munich, Germany). HBLAK was cultured in CnT-Prime Epithelial Culture Medium (CELLnTEC). All cells were incubated at 37 °C in a humidified atmosphere with 5% CO_2_.

### Reagents and siRNA transfection

Gemcitabine, AZD7762, Gö6976, romidepsin and givinostat were purchased from Selleck Chemicals. Gemcitabine was dissolved in water, all other compounds were dissolved in dimethyl sulfoxide (DMSO). Cisplatin solution was purchased from Accord Healthcare (London, UK). The reagents were stored at −70 °C until use.

### Viability assay

Viability was measured by the NAD(P)H-dependent MTT (3-(4,5-dimethylthiazol-2-yl)-2,5-diphenyltetrayolium bromide) dye reduction assay (Sigma Aldrich). Cells were seeded at a density of 3 × 10^3^ cells per well on 96-well culture plates, allowed to attach for 24 h, treated with different concentrations of gemcitabine and/or AZD7762 or other compounds and incubated for an additional 24 or 48 h, as indicated.

For siRNA-mediated knockdown, UCCs were transfected with 10 nmol/L CHK1 and/or CHK2 ON-TARGET plus Human siRNA or an ON-TARGET plus Non-targeting Pool siRNA for 24 h using Lipofectamine RNAi MAX (Invitrogen, Life Technologies, Darmstadt, Germany). Transfected cells were then harvested, seeded at a density of 3 × 10^3^ cells per well on 96-well culture plates, allowed to attach for 24 h, and treated with different concentrations of gemcitabine for 48 h. Cell viability was evaluated by MTT assay as described above.

### ATP-based cell viability and apoptosis assays

Cell viability was additionally measured via total cellular ATP as an indicator for viable cells using the CellTiter-Glo Luminescent Cell Viability Assay (Promega, Mannheim, Germany). After treatment with different concentrations of gemcitabine and/or AZD7762, cells were grown for 24 or 48 h and viability was measured by transferring cell aliquots into 96-well plates using CellTiter-Glo Reagent. Apoptosis induction after the treatment was quantified by the caspase-Glo 3/7 assay (Promega) and normalized to the ATP assay results.

### Colony forming assay and Giemsa-staining

For the colony forming assay, 3 × 10^3^ cells were seeded per 6 cm plate. After 10 days, cells were washed with PBS (Biochrom, Merck Millipore, Berlin, Germany), fixed in methanol, and stained with Giemsa solution (Merck, Darmstadt, Germany).

### Flow cytometry

For flow cytometry to evaluate changes in the cell cycle and apoptosis, 7.5 × 10^4^ cells were seeded in a 6-well culture plate 1 day prior to treatment with different concentrations of gemcitabine and/or AZD7762 for 24 or 48 h. Then, attached and floating cells were stained with PI-buffer containing 50 μg/ml propidium iodide, 0.1% sodium citrate and 0.1% Triton X-100, and flow cytometry was performed using a Miltenyi MACSQuant Analyzer (Milteny Biotec GmbH, Bergisch Gladbach, Germany). DNA histograms were fitted according to established protocols [21] using the MACSQuantify software (Miltenyi Biotec). Intervals for the respective cell cycle phases were individually configured for each DMSO treated control sample after 24 and 48 h, respectively, and applied to the corresponding drug-treated samples.

### Immunofluorescence stainings

For immunofluorescence, cells cultured on coverslips were treated as indicated for 24 or 48 h. Cells were fixed with 4% formaldehyde and permeabilized with 0.3% Triton X-100 in PBS for 10 min at room temperature (RT). Blocking was done in 10% goat serum (DAKO, Glostrup, Denmark), 0.3 M glycine and 0.1% Triton X100 in PBS for 1 h at RT. The fixed cells were incubated overnight at 4 °C with the primary antibodies pH2A.X (1:100, Cell Signaling Technology, Inc., Danvers, MA, USA) and 53-BP1 (1:250, clone BP18, Merck Millipore). Second antibodies were Alexa Fluor 488-conjugated goat-anti-rabbit IgG antibody and TRITC-conjugated goat-anti-mouse IgG (H + C) (Invitrogen, Life Technology) for 1 h at RT. Nuclei were counterstained with 500 ng/ml DAPI (4’,6-diamidino-2-phenylindole) for 5 min before mounting with fluorescence mounting medium (DAKO). Samples were imaged with a Nikon Eclipse 400 microscope.

### Western blotting

For western blotting, cells were maintained under the indicated conditions for 24 or 48 h and total protein lysates were prepared with RIPA-buffer containing 150 mM NaCl, 1% Triton X-100, 0.5% deoxycholate, 1% Nonidet P-40, 0.1% SDS, 1 mM EDTA, 50 mM Tris (pH 7.6) and 10 μl/ml protease inhibitor cocktail (Sigma Aldrich) for 30 min on ice. Protein concentrations were determined by BCA protein assay (Thermo Scientific, Rockford, IL, USA). Equal amount of proteins were separated by sodium dodecyl sulfate-polyacrylamide gel electrophoresis and transferred to PVDF membranes (Merck Millipore). Membranes were blocked with 5% bovine serum albumin or 5% non-fat milk in TBST (150 mM NaCl, 10 mM Tris, pH 7.4 and 0.1% Tween-20), washed and incubated with primary antibodies at RT for 1 h or at 4 °C overnight. Primary antibodies were used against phosphorylated ATM (Ser1981) (1:10000, Abcam, Cambridge, UK), ATM (1:1000, Cell Signaling Technology), phosphorylated ATR (Ser428) (1:1000, Cell Signaling Technology), ATR (1:1000, Cell Signaling Technology), phosphorylated CHK1 (Ser345) (1:1000, Cell Signaling Technology), CHK1(1:1000, Cell Signaling Technology), phosphorylated CHK2 (Thr68) (1:1000, Cell Signaling Technology), CHK2 (1:1000, Cell Signaling Technology), p21^CIP1^ (1:250, Santa Cruz Biotechnology, Heidelberg, Germany), poly [ADP-ribose] polymerase 1 (PARP1) (1:1000, Cell Signaling Technology), cleaved PARP (1:1000, Cell Signaling Technology), and anti-α-Tubulin B-512 (1:10000, Sigma Aldrich) or GAPDH (1:10000, Abcam) as loading controls. Secondary antibodies were HRP-conjugated goat-anti-mouse antibody and HRP-conjugated goat-anti-rabbit antibody (1:5000) (Santa Cruz Biotechnology) for 1 h at RT. Bands were visualised by chemiluminescence with the ECL select luminescence kit (GE Healthcare, Piscataway, NJ, USA) or the WesternBright Quantum kit (Biozym, Hessisch Oldendorf, Germany).

### Statistical analysis

Combination indexes (CI) were calculated by the Chou-Talalay method using CalcuSyn software (Biosoft, Cambridge, UK).

## Results

### The combination of gemcitabine and AZD7762 significantly reduced the viability of UCCs

**Fig. 1:**
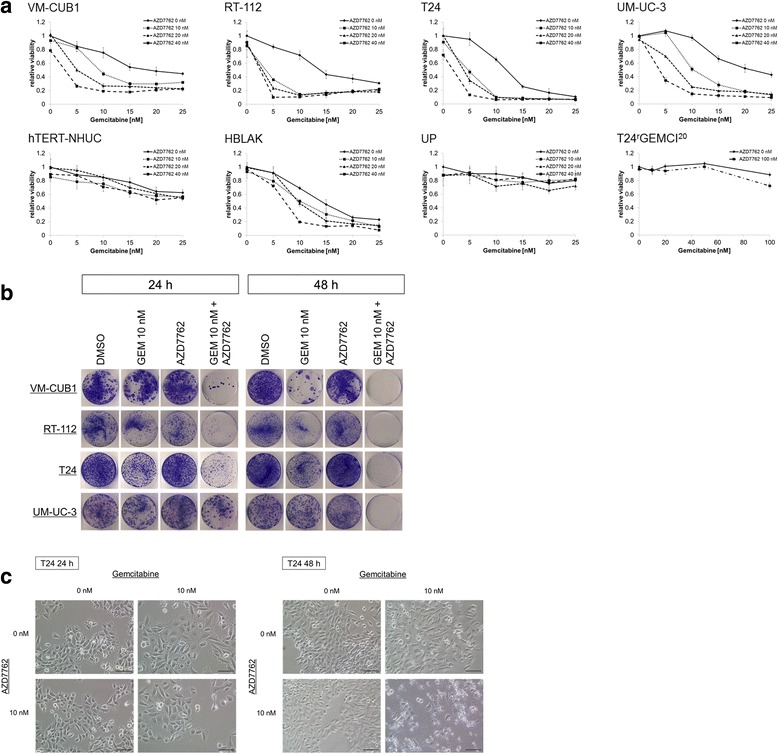
Viability and clonogenicity of UCCs and non-malignant urothelial cells after treatment with gemcitabine and/or AZD7762. **a** Relative cell viability in UCCs (VM-CUB1, RT-112, T24, UM-UC-3), non-malignant human urothelial cells (hTERT-NHU, HBLAK, UP) and T24^r^GEMCI^20^ cells was measured by MTT assay (mean ± SD, n = 4) after 48 h treatment with gemcitabine and/or AZD7762. **b** Giemsa staining of colonies from UCCs after 24 and 48 h treatments compared to DMSO solvent control. GEM stands for gemcitabine. The concentration of AZD7762 is 10 nM in RT-112 and T24 cells, and 20 nM in VM-CUB1 and UM-UC-3 cells. **c** Photomicrographs showing characteristic morphological changes in T24 cells treated with gemcitabine and AZD7762 (24 and 48 h) (**c**). Scale bar = 100 μm

In MTT assays, up to 40 nM AZD7762 had little effect of its own, but enhanced the effects of gemcitabine on the viability of four different urothelial carcinoma cell lines. However, it did not enhance the effect of gemcitabine on the non-malignant cell lines hTERT-NHUC and HBLAK, on normal urothelial (UP) cells, or the gemcitabine-resistant cell line T24^r^GEMCI^20^ (Fig. 1a). The combination efficiently inhibited the growth of UCCs in a dose-dependent manner. Combination index calculation demonstrated that the combined effect on cell viability of UCCs was synergistic (CI <1) under most treatment conditions (Table 1).

**Table 1:** Combination indexes

	AZD7762 (nM)		
Gemcitabine (nM)	10	20	40
VM-CUB1			
10	0.427	0.239	0.178
20	0.505	0.408	0.366
RT-112			
10	0.277	0.262	0.232
20	0.628	0.628	0.645
T24			
10	0.423	0.422	0.369
20	0.762	0.764	0.751
UM-UC-3			
10	0.495	0.392	0.343
20	0.725	0.718	0.639

Combination indexes (CI) calculated for the combination of gemcitabine and AZD7762 in urothelial carcinoma cells (CI < 1 indicates synergy)

We then investigated whether the combination of gemcitabine and AZD7762 also affects the clonogenicity of urothelial carcinoma cells. The combination treatment inhibited colony formation almost completely in UCCs whereas gemcitabine or AZD7762 alone inhibited colony formation only to a limited extent (Fig. 1b). Thus, the combination of gemcitabine with AZD7762 efficiently inhibited long-term growth of urothelial carcinoma cells in vitro.

Light microscopic examinations revealed characteristic morphological changes in UCCs treated with gemcitabine and AZD7762. After 24 h of gemcitabine single treatment or the combination treatment, cell size increased and cells became flatter compared with those treated with DMSO or AZD7762 alone. Cell structures were destroyed after 48 h of the combination treatment, whereas only minor further morphological changes occurred between 24 and 48 h of gemcitabine single treatment (Fig. 1c).

To compare the response of UCCs to sequential vs. simultaneous treatment, UCCs were pretreated with AZD7762 for 24 h and then incubated with gemcitabine for 48 h. Pretreatment with AZD7762 still enhanced the cytotoxic effect of gemcitabine, but not as strongly as simultaneous treatment (Additional file 1: Figure S1).

MTT assays were also employed to investigate whether AZD7762 likewise enhanced the activity of cisplatin and the class I HDAC-specific inhibitors, romidepsin and givinostat, three agents highly toxic to UC cells. Cisplatin produces mostly intrastrand DNA cross-links [22]. Pharmacologic inhibition of HDAC1 and HDAC2 by romidepsin or givinostat severely disrupts cell cycle progression and likewise induces DNA damage [18]. However, none of these agents acted synergistically with AZD7762 (Additional file 2: Figure S2a).

### AZD7762 enhances gemcitabine activity through CHK1 inhibition

Treatment with gemcitabine or cisplatin, but not with romidepsin or givinostat strongly enhanced CHK1 phosphorylation at Ser345 (Additional file 2: Figure S2b), indicating that the extent of CHK1 phosphorylation does not predict whether AZD7762 sensitises cells to a DNA-damaging compound in a synergistic manner.

**Fig. 2:**
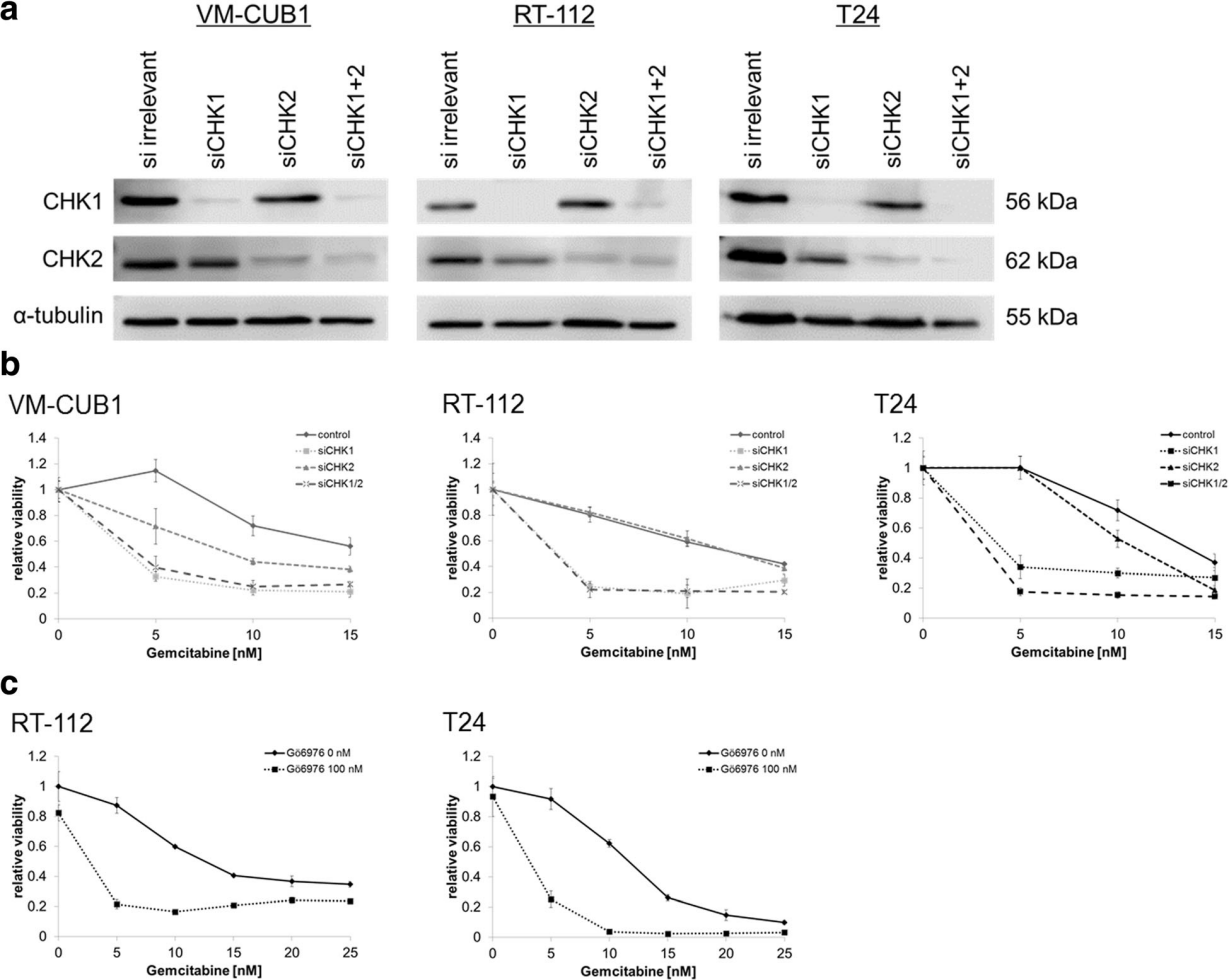
Effects of siRNA mediated knockdown of CHK1, CHK2 or CHK1/2 on sensitivity to gemcitabine. **a** Effects of specific CHK1, CHK2, or both siRNAs compared to control siRNA (irrelevant) on CHK1 and CHK2 protein expression after 48 h transfection in VM-CUB1, RT-112 and T24 cells. **b** Effect of gemcitabine on cell viability in VM-CUB1, RT-112 and T24 cell lines after siRNA knockdown of CHK1, CHK2 or both CHK1/2 compared to irrelevant control. Note that for each siRNA treatment mean viability without gemcitabine was set as 1. **c** Relative cell viability in RT-112 and T24 cells was measured by MTT assay (mean ± SD, n = 4) after 48 h treatment with gemcitabine and/or Gö6976

To investigate the relative contributions of inhibition of CHK1 or CHK2 to gemcitabine treatment, we used siRNA to selectively deplete CHK1 or CHK2 from UCC cell lines. Compared to nonspecific siRNA-treated cells, CHK1-depleted cells were efficiently sensitised to gemcitabine, whereas CHK2-depleted cells were not markedly sensitised (Fig. 2a, b). Additional depletion of CHK2 increased the sensitisation induced by CHK1 depletion. These data suggest that sensitisation to gemcitabine by AZD7762 is mainly mediated by CHK1 inhibition and CHK2 inhibition has a supplementary role. Accordingly, pharmacological inhibition of CHK1 by the CHK1-specific inhibitor Gö6976 also efficiently sensitised T24 cells to gemcitabine (Fig. 2c).

### The combination of gemcitabine and AZD7762 causes cell accumulation in S-phase and apoptosis

**Fig. 3:**
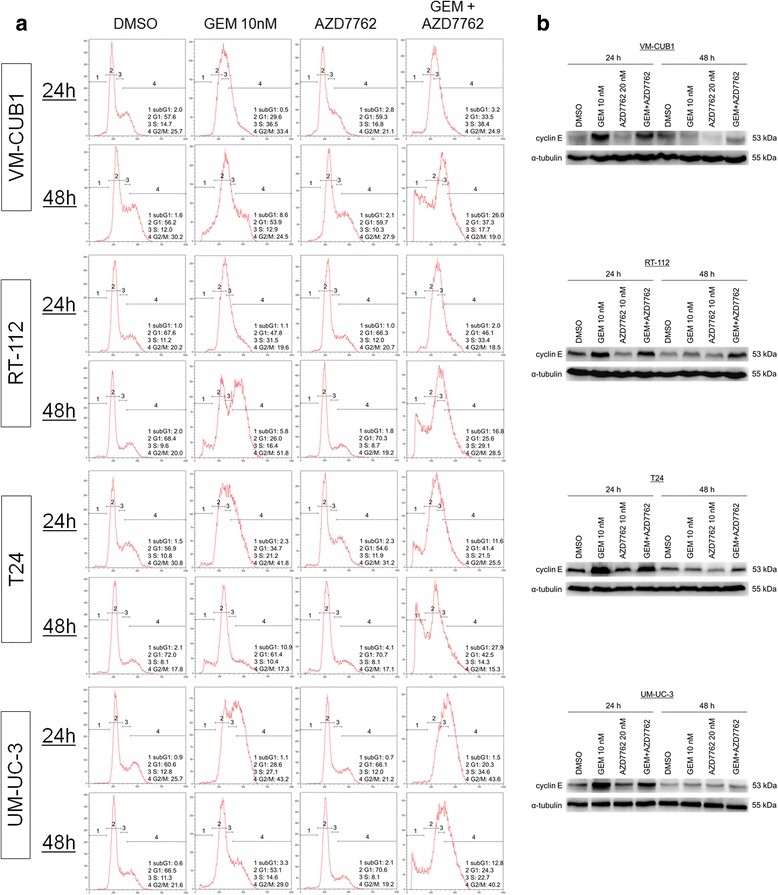
Effects of gemcitabine and AZD7762 on cell cycle distribution. **a** UCCs were treated for 24 or 48 h with gemcitabine (10 nM) and/or AZD7762 (10 nM in RT-112 and T24 cells, and 20 nM in VM-CUB1 and UM-UC-3 cells) before cell cycle distribution was analysed by flow cytometry. DMSO served as a solvent control. **b** Cyclin E protein expression subsequent to gemcitabine and/or AZD7762 treatment was evaluated by Western blot analysis

To further investigate the mechanisms by which AZD7762 increases the effects of gemcitabine, we analysed cell cycle distribution and apoptosis induction in response to treatment. After 24 h, UCCs treated with gemcitabine alone or with the combination displayed accumulation of cells with S-phase DNA content (Fig. 3a). However, during gemcitabine single treatment many cells appeared to overcome this accumulation after 48 h of treatment. In contrast, cells treated with gemcitabine and AZD7762 remained accumulated in S phase or underwent apoptosis, as indicated by a significant increase in the fraction of sub-G1 cells in all cell lines after 48 h combined treatment, compared with untreated controls or each single treatment. In the T24 cell line, the increase in the fraction of sub-G1 cells was remarkable already after 24 h.

Consistently with the flow cytometry data, Western blot analysis showed that the expression of cyclin E, which is maximal in S-phase, was increased after 24 h of gemcitabine treatment in UCCs but returned to control levels after 48 h (Fig. 3b). However, when gemcitabine was combined with AZD7762, its expression remained elevated after 48 h in VM-CUB1, RT-112, and T24 cells.

### The combination of gemcitabine and AZD7762 induced apoptosis in UCCs

**Fig. 4:**
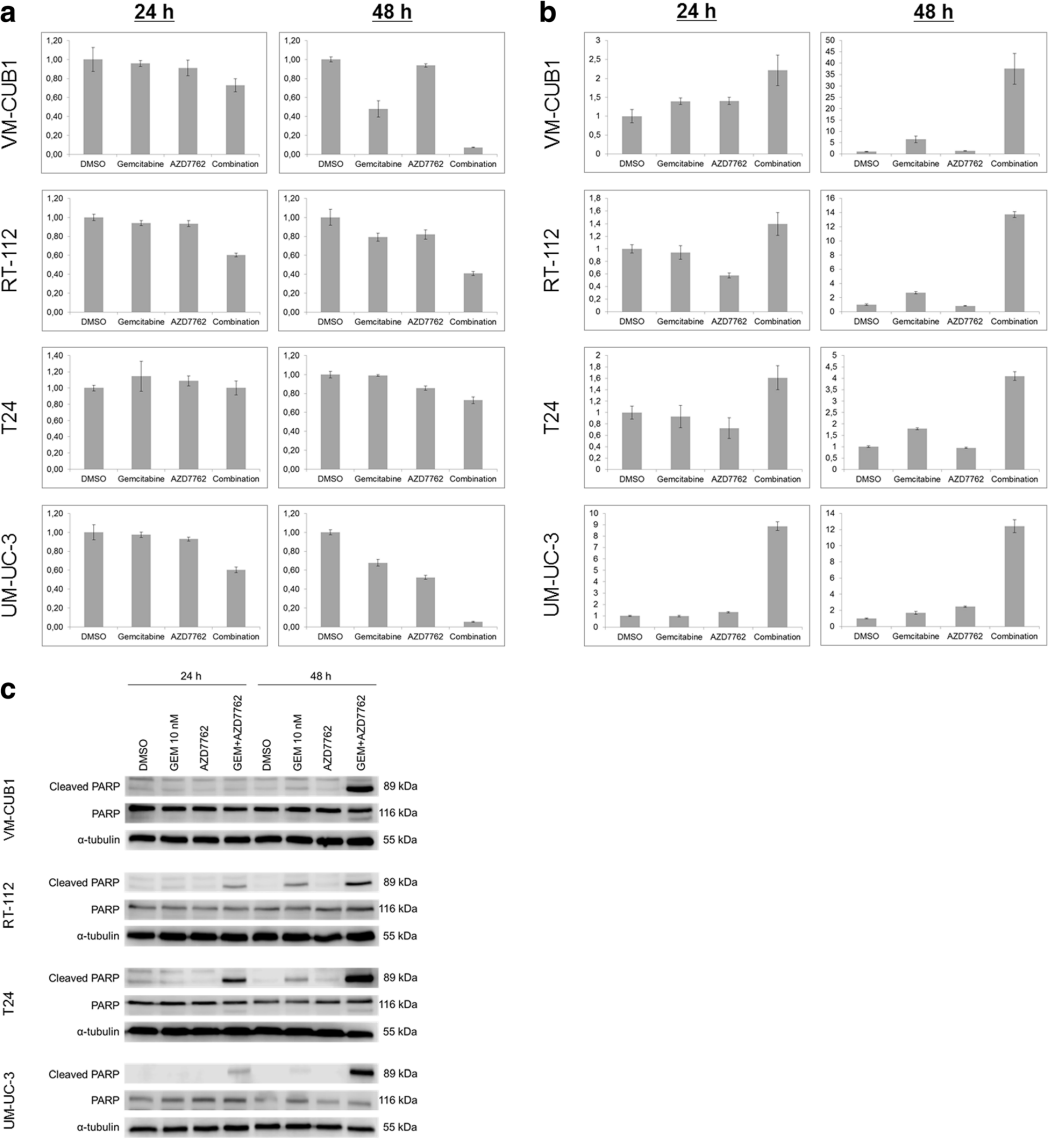
Analysis of cell death mechanism after treatments. **a** Relative cell viability in UCCs was measured after treatments with 10 nM gemcitabine and/or AZD7762 using the CellTiterGlo ATP-assay. The concentration of AZD7762 used was 10 nM in RT-112 and T24 cells, and 20 nM in VM-CUB1 and UM-UC-3 cells. **b** Induction of apoptosis was measured by caspase 3/7 assay. **c** Cleaved PARP was measured after the indicated treatments (24 and 48 h)

After treatment with different concentrations of gemcitabine and/or AZD7762, UCC viability was measured additionally by assaying ATP. The combination treatment significantly decreased cell viability in all UCCs (Fig. 4a). Significantly increased caspase-3/7 activity was observed after 48 h of combination treatment in all cell lines, most strongly in RT-112 (Fig. 4b). Accordingly, increased levels of cleaved PARP were detected by Western blot analysis following combination treatment (Fig. 4c). In the T24 cell line, cleaved PARP already increased after 24 h of treatment, fitting the advanced increase in the sub-G1 fraction in cell cycle analysis. Thus, the combination of gemcitabine and AZD7762 efficiently induced apoptosis in all UCCs, albeit with somewhat different time courses.

### AZD7762 inhibited DNA damage repair sensitising UCCs to gemcitabine-induced DNA damage

**Fig. 5:**
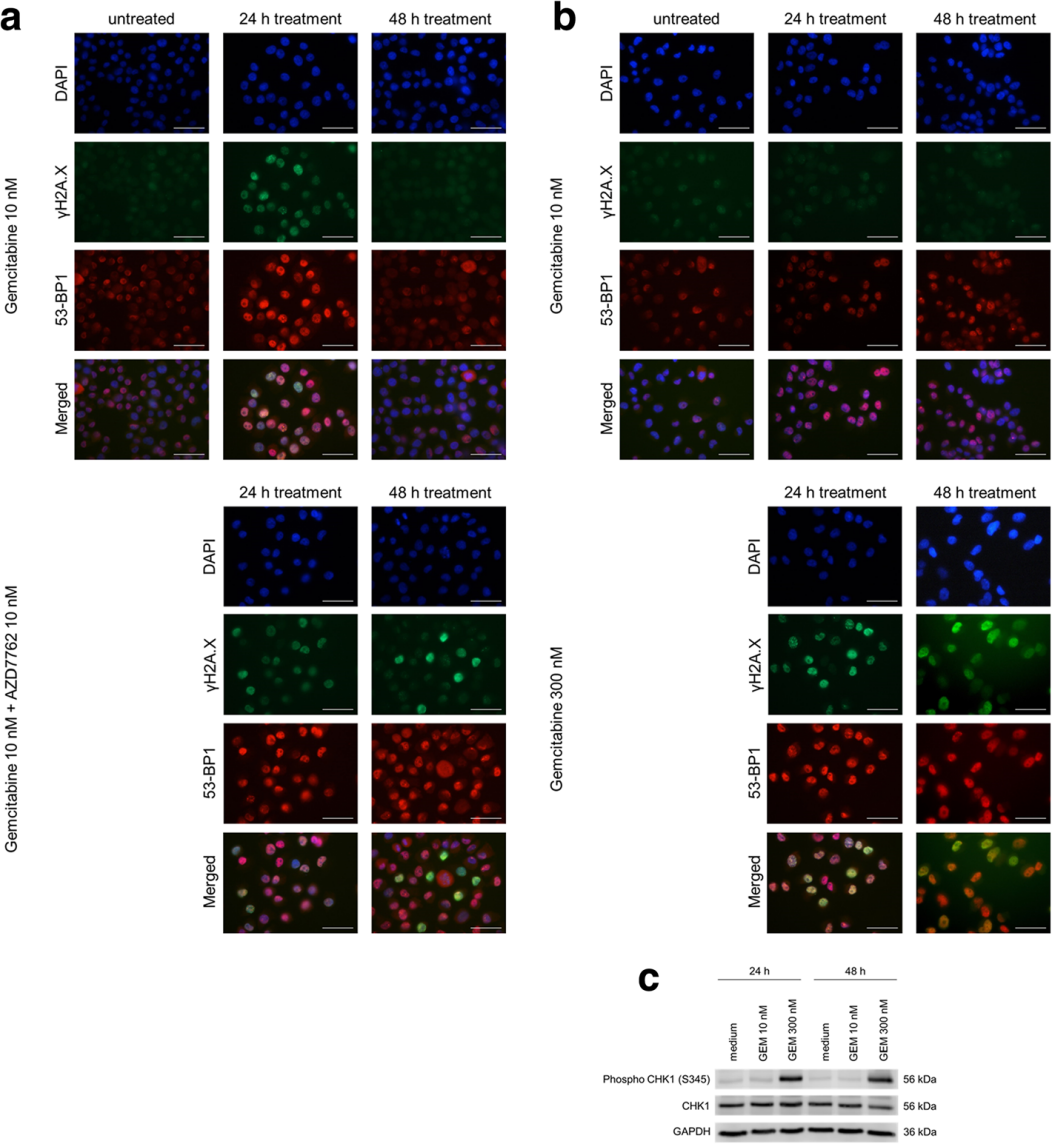
Immunofluorescent analysis of gemcitabine-induced γH2A.X and 53-BP1 focus formation. **a**, **b** Immunofluorescence staining of γH2A.X (*green*), 53-BP1 (*red*), and nuclei staining with DAPI (*blue*) in T24 (a) and T24^r^GEMCI^20^ (b) cells after the indicated treatments. Scale bar = 50 μm. **c** Western blot analysis of S345 CHK1 after treatments with gemcitabine in T24^r^GEMCI^20^ cells (24 and 48 h). As loading control, GAPDH was stained

To investigate the presence of unrepaired double strand breaks (DSB) induced by gemcitabine and AZD7762, we assessed colocalisation of double staining for the DSB markers γH2A.X and 53-BP1 [23] by immunofluorescence (Fig. 5a, Additional file 3: Figure S3). As anticipated, exposure of UCC cells to gemcitabine elevated γH2A.X and 53-BP1 focal staining after 24 h of treatment. Staining returned to near control levels after 48 h, indicating that the DNA DSBs were repaired. Addition of AZD7762 with gemcitabine increased γH2A.X and 53-BP1 levels after 24 h, and this increase was remarkably prolonged until after 48 h. These results indicate that AZD7762 disturbed gemcitabine-induced DNA damage repair.

In the T24^r^GEMCI^20^ cell line, 10 nM gemcitabine did not elevate γH2A.X and 53-BP1 foci, whereas 300 nM concentration, which is much higher than clinically achievable, led to a sustained increase (Fig. 5b). These data suggest that the repair of DNA DSBs was delayed or abrogated in the gemcitabine-resistant cell line at very high concentrations. We furthermore analysed the response to gemcitabine in the T24^r^GEMCI^20^ cell line by Western blot analysis. The ATR-mediated phosphorylation of CHK1 (Ser345 CHK1) was increased only at the higher concentration of gemcitabine and likewise sustained after 48 h of treatment (Fig. 5c).

**Fig. 6:**
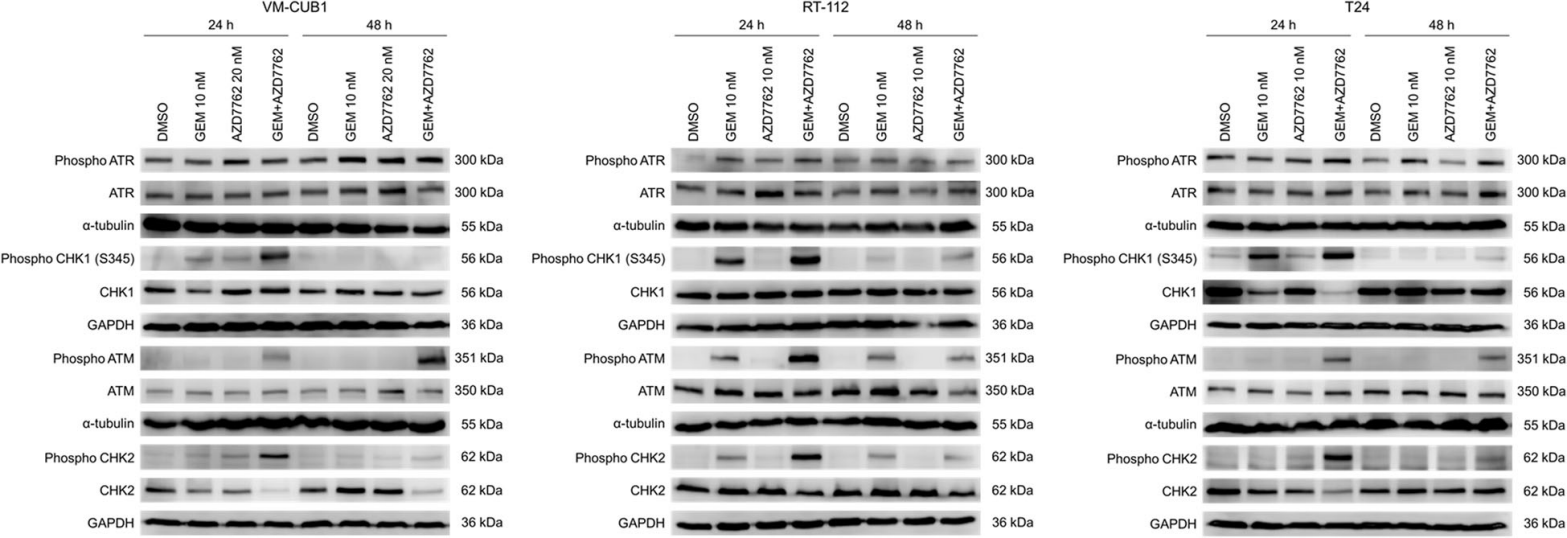
Western blot analysis of checkpoint factors. Whole cell lysates from UCCs treated with gemcitabine (10 nM) and/or AZD7762 (10 or 20 nM) for 24 or 48 h were assayed for the indicated proteins or their phosphorylation. As loading controls, GAPDH or α-tubulin were stained on each blot

To confirm that AZD7762 inhibits CHK1/CHK2 in UCCs, CHK1/CHK2 signalling was investigated. UCC cultures were treated with gemcitabine in the presence or absence of AZD7762 for 24 and 48 h before harvesting for Western blot analysis (Fig. 6). Ser345 CHK1 was transiently phosphorylated after 24 h of gemcitabine treatment returning to near control levels after 48 h. Ser345 CHK1 phosphorylation was further enhanced when UCCs were co-treated with AZD7762. ATM became phosphorylated (at Ser1981) after 24 h of gemcitabine exposure and ATM phosphorylation was increased when gemcitabine was combined with AZD7762. Phosphorylation of CHK2 at Thr68, affected by activated ATM, was also detected after 24 h of combination treatment. The increased activation of checkpoint kinases is consistent with more pronounced DNA damage upon co-administration of AZD7762 with gemcitabine. In the RT-112 cell line, ATM and CHK2 phosphorylation increased even after 48 h of gemcitabine single treatment suggesting that gemcitabine-induced double strand breaks persisted even after 48 h in this cell line.

### Relation of sensitisation by AZD7762 to p21^CIP1^ expression

In The Cancer Genome Atlas (TCGA) dataset, 14% of invasive bladder cancers have mutations in *CDKN1A* encoding the cyclin-dependent kinase inhibitor p21^CIP1^ [24]. It was previously reported that double mutant p53/p21-deficient bladder cancers were more sensitive to combined treatment with gemcitabine and a CHK inhibitor [25]. To examine this further, we performed Western blot analysis in the four UCCs used in the current study. Three expressed p21^CIP1^, whereas RT-112 cells lacked expression (Additional file 4: Figure S4a) due to a homozygous frame-shift mutation at codon 29 [26]. As mentioned above, in our hands, AZD7762 sensitised all four UCCs including RT-112 to gemcitabine in a synergistic fashion, although checkpoint activation by gemcitabine alone was more pronounced in RT-112. We therefore assessed the changes in the expression of p21^CIP1^. Expression of p21^CIP1^ increased in VM-CUB1 cells following treatment with gemcitabine or gemcitabine-AZD7762 combination, whereas p21^CIP1^ remained undetectable in RT-112 cells, as expected (Additional file 4: Figure S4b). These data suggest that sensitisation of UCCs to gemcitabine by AZD7762 is qualitatively independent of p21^CIP1^ expression.

## Discussion

In the present study, we showed that AZD7762, an ATP competitive inhibitor of checkpoint kinases, can strongly sensitise UCCs to the ribonucleotide reductase inhibitor gemcitabine. The effect of AZD7762 is associated with abrogation of the G2 checkpoint activation induced by gemcitabine and especially with persistence of unrepaired DNA damage, as indicated by our findings that AZD7762 increased ATR-mediated CHK1 phosphorylation (Ser345 CHK1) and that it inhibited the repair of gemcitabine-induced double strand breaks as evidenced by sustained expression of γH2A.X and 53-BP1. There are likely several reasons why AZD7762 leads to persistence of double strand breaks, including its inhibitory effects on Rad51 focus formation and homologous recombination DNA repair [27] and on the function of CHK1 in the maintenance of replication forks [28]. The enhancement of cytotoxicity by AZD7762 was relatively specific to gemcitabine, as the combination effect was weaker with other compounds causing DNA strand-breaks, like cisplatin or HDAC1/2 inhibitors (Additional file 2: Figure S2a).

As AZD7762 is an equally potent inhibitor of both CHK1 and CHK2 [14], a priori, inhibition of both kinases might contribute to its enhancement of gemcitabine activity on UCCs. Indeed, CHK2 is also capable of arresting the cell cycle by several mechanisms [29]. However, siRNA depletion experiments showed that interference with CHK1 results in a much more pronounced UCC sensitisation to gemcitabine compared to interference with CHK2, but that depletion of both kinases was most efficient. Therefore, interference with CHK1 is primarily responsible for UCC sensitisation to gemcitabine. In concordance, pharmacological inhibition of CHK1 by the CHK1-specific inhibitor Gö6976 [30] also efficiently sensitised UCCs to gemcitabine. However, the effects of CHK1 depletion are further enhanced by additional inhibition of CHK2 activity. Notably, although *CHK1* gene knock-out is lethal in embryos [31] and induces apoptosis in embryonic stem cells [32], the depletion of CHK1 by siRNA in somatic cells on its own has been reported to cause little cytotoxicity and enhance the efficacy of DNA-damaging drugs in p53-deficient cancer cell lines [33]. In accordance, we did not find AZD7762 to sensitise non-cancerous cells to gemcitabine. Taken together, these data suggest that selective CHK1 inhibition may potentiate the cytotoxicity of gemcitabine selectively in tumour cells. Reasons for this selectivity may include differences in checkpoint function [34, 35] and p53 regulation [36] between normal cells and cancer cells. Tumour cells harbouring defects in p53 function lack an efficient G1 checkpoint and thus have to rely on the S or G2 checkpoints for DNA repair, in which CHK1/2 have crucial functions [36, 37]. Checkpoint abrogation can therefore promote DNA-damage-induced mitotic catastrophe and cell death in p53-defective tumour cells [38], whereas normal cells may tolerate DNA damage stress by activating the G1 checkpoint through normal p53 function [35, 36].

AZD7762 did not resensitise gemcitabine-resistant T24^r^GEMCI^20^ cells to gemcitabine when administered at clinically achievable concentrations. Neither γH2A.X and 53-BP1 focus formation nor phosphorylation of CHK1 was increased in T24^r^GEMCI^20^ cells by the investigated gemcitabine concentrations, indicating a lack of replication stress and, hence, of the target of CHK1 inhibition. Accordingly, in a phase I trial conducted in patients with solid tumours - other than UC - only gemcitabine-naïve patients responded to the combination of AZD7762 and gemcitabine [39].

Several findings indicate that p21^CIP1^ is involved in the control of DNA repair pathways including homologous recombination (HR) [40]. p21^CIP1^ promotes HR by inhibiting cyclin dependent kinases (CDK), since increased CDK activity in the absence of p21^CIP1^ is associated with elevated levels of DNA damage [41]. It has accordingly been reported that p53/p21^CIP1^ dual-mutant UCCs display an enhanced cytotoxic response to genotoxic agents combined with CHK1 inhibition over either p53- or p21^CIP1^- deficient cells [25]. Indeed, in the RT-112 cell line, which has an inactivating mutation in p21^CIP1^, gemcitabine-induced double strand breaks persisted longer (Fig. 6) and the sensitisation to gemcitabine by AZD7762 appeared to be quantitatively stronger (Fig. 1a) than in other cell lines. This observation suggests a role for p21^CIP1^ in DNA repair in UCCs as well. In the current study, however, CHK1 inhibition by AZD7762 sensitised UCCs to gemcitabine regardless of their p21^CIP1^ status and in the T24 cell line AZD7762 abrogated DNA repair of gemcitabine-induced DNA double strand breaks (Fig. 5a). These observations may mean that AZD7762 overcomes the influence of p21^CIP1^ on the DNA repair process.

A phase I clinical trial of AZD7762 has been conducted in patients with solid tumours other than urothelial carcinoma but was prematurely terminated due to cardiac toxicity [39]. Currently, novel inhibitors specific for CHK1 or with broader target specificity are tested in several clinical trials [42, 43]. In combination with gemcitabine, such compounds might prove efficacious with tolerable toxicity in patients with treatment-naïve advanced bladder cancers who are ineligible for cisplatin.

## Conclusions

The CHK1/2 inhibitor AZD7762 enhanced gemcitabine activity in gemcitabine-sensitive UCCs through inhibition of the gemcitabine-induced DNA damage response, which is predominantly mediated through CHK1 inhibition. Hence, the current study provides a rationale for the testing of CHK1 inhibitors or other therapy strategies that interfere with CHK1 function in combination with gemcitabine in gemcitabine-naïve UC patients, in particular for patients with advanced and/or metastatic disease who are ineligible for cisplatin. Notably, potentiation of gemcitabine effects by AZD7762 in xenografts from several other cancer types has been already reported [14, 44, 45]. The next step towards application of our results in bladder cancer should therefore be determining the optimal combinations of gemcitabine and CHK1 inhibitors in animal models of the disease.

## Additional files

Additional file 1: Figure S1. Viability in UCCs after sequential treatment with AZD7762 and gemcitabine. Relative cell viability in several UCCs was measured by MTT assay (mean ± SD, n = 4) after cells were sequentially treated (pretreated with AZD7762 for 24 h and then incubated with gemcitabine for 48 h). (TIF 142 kb)
Additional file 2: Figure S2. Viability in UCCs after combined treatments with cisplatin, romidepsin or givinostat, and AZD7762. a Relative cell viability in the T24 and RT-112 cell lines was measured by MTT assay (mean ± SD, n = 4) after cells were treated for 48 h either with cisplatin, romidepsin or givinostat combined with AZD7762. b Western blot analysis of S345 CHK1 after treatments with gemcitabine (10 nM), cisplatin (5 μM), romidepsin (4 nM) and givinostat (0.5 μM) in T24 cells (24 and 48 h). As loading control, GAPDH was stained. (TIF 214 kb)
Additional file 3: Figure S3.
**a**, **b** Immunofluorescence staining of γH2A.X (green), 53-BP1 (red), and nuclei staining with DAPI (blue) in VM-CUB1 (**a**) and RT-112 (**b**) cells after the indicated treatments. Scale bar = 50 μm. (TIF 1728 kb)
Additional file 4: Figure S4.
**a** CDKN1A mutations in UCCs. p21^CIP1^ expression of UCCs was assessed by western blotting with α-tubulin as loading control. **b** Induction of p21^CIP1^ was assessed by western blot analysis after 24 or 48 h of treatment with gemcitabine and/or AZD7762 in CDKN1A-wildtype VM-CUB1 and CDKN1A-mutant RT-112 cells. (TIF 126 kb)

## Abbreviations

ATM: Ataxia telangiectasia mutated
ATR: Ataxia telangiectasia and RAD3-related
CDK: Cyclin dependent kinase
CHK: Checkpoint kinase
DSB: Double strand break
MTT: 3-(4,5-dimethylthiazol-2-yl)-2,5-diphenyltetrayolium bromide
UCC: Urothelial carcinoma cell line

## References

[1] Knowles MA, Hurst CD (2015). Molecular biology of bladder cancer: new insights into pathogenesis and clinical diversity. Nat Rev Cancer.

[2] Carballido EM, Rosenberg JE (2014). Optimal treatment for metastatic bladder cancer. Curr Oncol Rep.

[3] Milowsky MI, Rumble RB, Booth CM, Gilligan T, Eapen LJ, Hauke RJ (2016). Guideline on Muscle-Invasive and Metastatic Bladder Cancer (European Association of Urology guideline): American Society of Clinical Oncology Clinical Practice Guideline Endorsement. J Clin Oncol.

[4] Von der Maase H, Sengelov L, Roberts JT, Ricci S, Dogliotti L, Oliver T (2005). Long-term survival results of a randomized trial comparing gemcitabine plus cisplatin, with methotrexate, vinblastine, doxorubicin, plus cisplatin in patients with bladder cancer. J Clin Oncol.

[5] Dash A, Galsky MD, Vickers AJ, Serio AM, Koppie TM, Dalbagni G (2006). Impact of renal impairment on eligibility for adjuvant cisplatin-based chemotherapy in patients with urothelial carcinoma of the bladder. Cancer.

[6] Witjes JA, Compérat E, Cowan NC, De Santis M, Gakis G, Lebret T (2014). EAU guidelines on muscle-invasive and metastatic bladder cancer: summary of the 2013guidelines. Eur Urol.

[7] De Santis M, Bellmunt J, Mead G, Kerst JM, Leahy M, Maroto P (2012). Randomized phase II/III trial assessing gemcitabine/carboplatin and methotrexate/carboplatin/vinblastine in patients with advanced urothelial cancer who are unfit for cisplatin-based chemotherapy: EORTC study 30986. J Clin Oncol.

[8] Lord CJ, Ashworth A (2012). The DNA damage response and cancer therapy. Nature.

[9] Sancar A, Lindsey-Boltz LA, Unsal-Kaçmaz K, Linn S (2004). Molecular mechanisms of mammalian DNA repair and the DNA damage checkpoints. Annu Rev Biochem.

[10] Lee JH, Paull TT (2005). ATM activation by DNA double-strand breaks through the Mre11-Rad50-Nbs1 complex. Science.

[11] Zou L, Elledge SJ (2003). Sensing DNA damage through ATRIP recognition of RPA-ssDNA complexes. Science.

[12] Bartek J, Falck J, Lukas J (2001). CHK2 kinase--a busy messenger. Nat Rev Mol Cell Biol.

[13] Bartek J, Lukas J (2003). Chk1 and Chk2 kinases in checkpoint control and cancer. Cancer Cell.

[14] Zabludoff SD, Deng C, Grondine MR, Sheehy AM, Ashwell S, Caleb BL (2008). AZD7762, a novel checkpoint kinase inhibitor, drives checkpoint abrogation and potentiates DNA-targeted therapies. Mol Cancer Ther.

[15] Huang P, Chubb S, Hertel LW, Grindey GB, Plunkett W (1991). Action of 2’,2’-difluorodeoxycytidine on DNA synthesis. Cancer Res.

[16] Heinemann V, Xu YZ, Chubb S, Sen A, Hertel LW, Grindey GB (1992). Cellular elimination of 2’,2’-difluorodeoxycytidine 5’-triphosphate: a mechanism of self-potentiation. Cancer Res.

[17] Ferreira CG, Span SW, Peters GJ, Kruyt FA, Giaccone G (2000). Chemotherapy triggers apoptosis in a caspase-8-dependent and mitochondria-controlled manner in the non-small cell lung cancer cell line NCI-H460. Cancer Res.

[18] Pinkerneil M, Hoffmann MJ, Deenen R, Köhrer K, Arent T, Schulz WA (2016). Inhibition of class I histone deacetylases 1 and 2 promotes urothelial carcinoma cell death by various mechanisms. Mol Cancer Ther.

[19] Chapman EJ, Hurst CD, Pitt E, Chambers P, Aveyard JS, Knowles MA (2006). Expression of hTERT immortalises normal human urothelial cells without inactivation of the p16/Rb pathway. Oncogene.

[20] Vallo S, Michaelis M, Rothweiler F, Bartsch G, Gust KM, Limbart DM (2015). Drug-resistant urothelial cancer cell lines display diverse sensitivity profiles to potential second-line therapeutics. Transl Oncol.

[21] Ormerod MG. Flow cytometry: Royal Microscopical Society microscopy handbooks. 2nd ed. New York: Garland Science; 1999.

[22] Fichtinger-Schepman AM, van der Veer JL, den Hartog JH, Lohman PH, Reedijk J (1985). Adducts of the antitumor drug cis-diamminedichloroplatinum(II) with DNA: formation, identification, and quantitation. Biochemistry.

[23] Bonner WM, Redon CE, Dickey JS, Nakamura AJ, Sedelnikova OA, Solier S (2008). GammaH2AX and cancer. Nat Rev Cancer.

[24] The Cancer Genome Atlas Research Network (2014). Comprehensive molecular characterization of urothelial bladder carcinoma. Nature.

[25] Liu Y, Kwiatkowski DJ (2015). Combined CDKN1A/TP53 mutation in bladder cancer is a therapeutic target. Mol Cancer Ther.

[26] Forbes SA, Bindal N, Bamford S, Cole C, Kok CY, Beare D (2011). COSMIC: mining complete cancer genomes in the Catalogue of Somatic Mutations in Cancer. Nucleic Acids Res.

[27] Morgan MA, Parsels LA, Zhao L, Parsels JD, Davis MA, Hassan MC (2010). Mechanism of radiosensitization by the Chk1/2 inhibitor AZD7762 involves abrogation of the G2 checkpoint and inhibition of homologous recombinational DNA repair. Cancer Res.

[28] Sampath D, Rao VA, Plunkett W (2003). Mechanisms of apoptosis induction by nucleoside analogs. Oncogene.

[29] Zannini L, Delia D, Buscemi G (2014). CHK2 kinase in the DNA damage response and beyond. J Mol Cell Biol.

[30] Kohn EA, Yoo CJ, Eastman A (2003). The protein kinase C inhibitor Gö6976 is a potent inhibitor of DNA damage-induced S and G2 cell cycle checkpoints. Cancer Res.

[31] Takai H, Tominaga K, Motoyama N, Minamishima YA, Nagahama H, Tsukiyama T (2000). Aberrant cell cycle checkpoint function and early embryonic death in Chk1(−/−) mice. Genes Dev.

[32] Liu Q, Guntuku S, Cui XS, Matsuoka S, Cortez D, Tamai K (2000). Chk1 is an essential kinase that is regulated by Atr and required for the G2/M DNA damage checkpoint. Genes Dev.

[33] Chen Z, Xiao Z, Chen J, Ng SC, Sowin T, Sham H (2003). Human Chk1 expression is dispensable for somatic cell death and critical for sustaining G2 DNA damage checkpoint. Mol Cancer Ther.

[34] Bartkova J, Horejsí Z, Koed K, Krämer A, Tort F, Zieger K (2005). DNA damage response as a candidate anti-cancer barrier in early human tumorigenesis. Nature.

[35] Wang WT, Catto JW, Meuth M (2015). Differential response of normal and malignant urothelial cells to CHK1 and ATM inhibitors. Oncogene.

[36] Kawabe T (2004). G2 checkpoint abrogators as anticancer drugs. Mol Cancer Ther.

[37] Zhou BB, Bartek J (2004). Targeting the checkpoint kinases: chemosensitization versus chemoprotection. Nat Rev Cancer.

[38] Castedo M, Perfettini JL, Roumier T, Andreau K, Medema R, Kroemer G (2004). Cell death by mitotic catastrophe: a molecular definition. Oncogene.

[39] Sausville E, LoRusso P, Carducci M, Carter J, Quinn MF, Malburg L (2014). Phase I dose-escalation study of AZD7762, a checkpoint kinase inhibitor, in combination with gemcitabine in US patients with advanced solid tumors. Cancer Chemother Pharmacol.

[40] Dutto I, Tillhon M, Cazzalini O, Stivala LA, Prosperi E (2015). Biology of the cell cycle inhibitor p21(CDKN1A): molecular mechanisms and relevance in chemical toxicology. Arch Toxicol.

[41] Mauro M, Rego MA, Boisvert RA, Esashi F, Cavallo F, Jasin M (2012). p21 promotes error-free replication-coupled DNA double-strand break repair. Nucleic Acids Res.

[42] Hong D, Infante J, Janku F, Jones S, Nguyen LM, Burris H (2016). Phase I study of LY2606368, a checkpoint kinase 1 inhibitor, in patients with advanced cancer. J Clin Oncol.

[43] Daud AI, Ashworth MT, Strosberg J, Goldman JW, Mendelson D, Springett G (2015). Phase I dose-escalation trial of checkpoint kinase 1 inhibitor MK-8776 as monotherapy and in combination with gemcitabine in patients with advanced solid tumors. J Clin Oncol.

[44] Parsels LA, Qian Y, Tanska DM, Gross M, Zhao L, Hassan MC (2011). Assessment of chk1 phosphorylation as a pharmacodynamic biomarker of chk1 inhibition. Clin Cancer Res.

[45] Itamochi H, Nishimura M, Oumi N, Kato M, Oishi T, Shimada M (2014). Checkpoint kinase inhibitor AZD7762 overcomes cisplatin resistance in clear cell carcinoma of the ovary. Int J Gynecol Cancer.

