# Supplementary figures and images for: Checkpoint kinase inhibitor AZD7762 strongly sensitises urothelial carcinoma cells to gemcitabine
Source: J Exp Clin Cancer Res. 2017 Jan 3;36:1. doi: 10.1186/s13046-016-0473-1 (PMC5209915; doi:10.1186/s13046-016-0473-1)

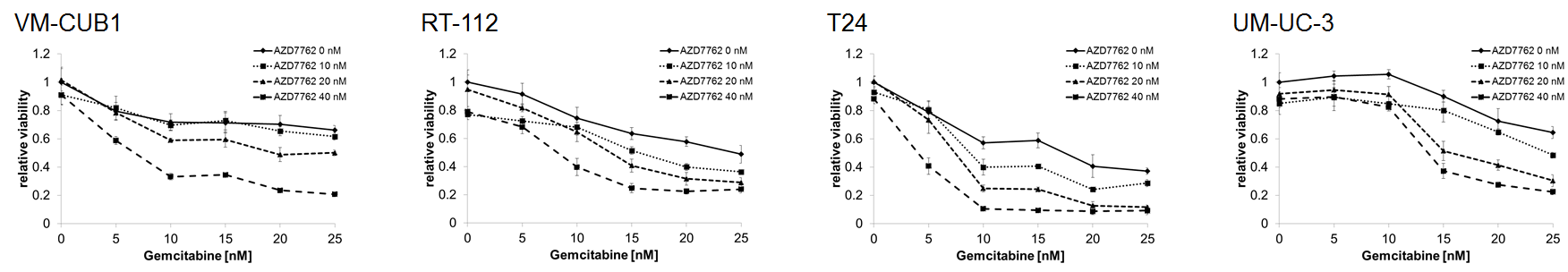

Supplement: Additional file 1: Figure S1. — Viability in UCCs after sequential treatment with AZD7762 and gemcitabine. Relative cell viability in several UCCs was measured by MTT assay (mean ± SD, n = 4) after cells were sequentially treated (pretreated with AZD7762 for 24 h and then incubated with gemcitabine for 48 h). (TIF 142 kb) [file 13046_2016_473_MOESM1_ESM.tif]

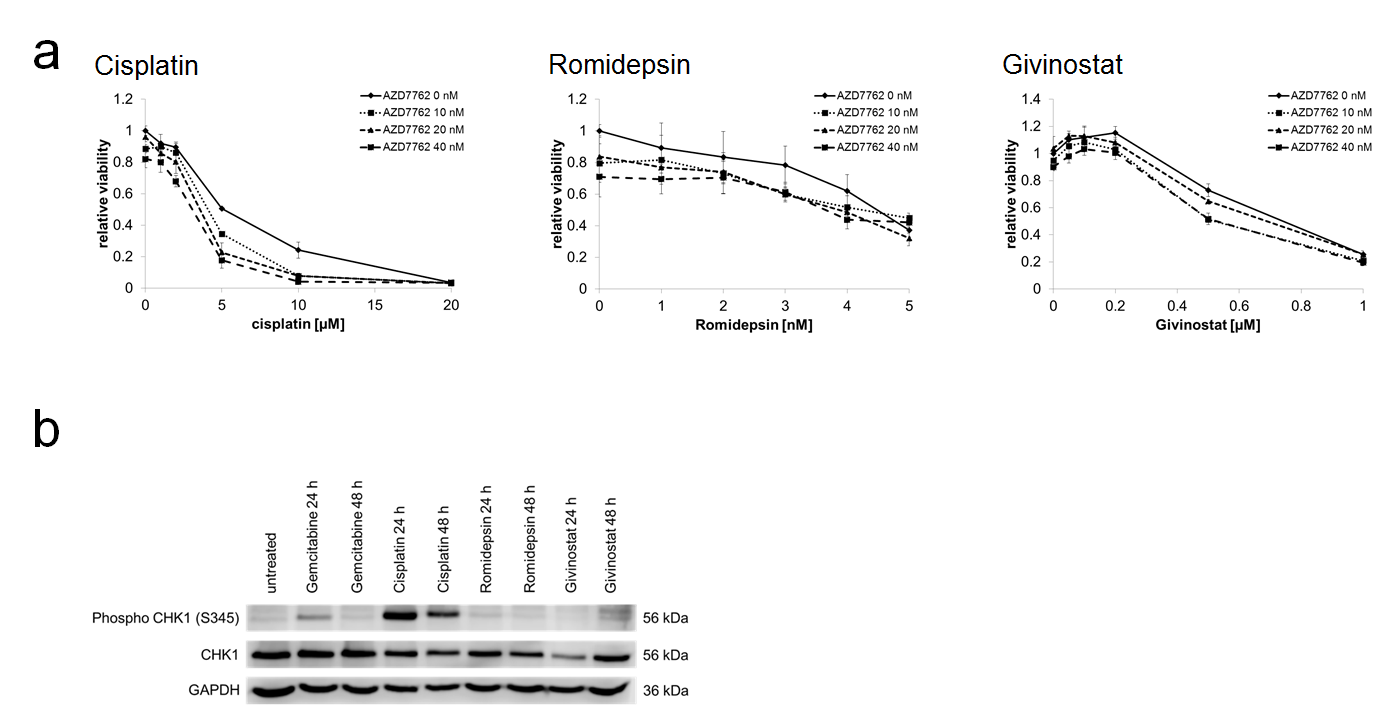

Supplement: Additional file 2: Figure S2. — Viability in UCCs after combined treatments with cisplatin, romidepsin or givinostat, and AZD7762. a Relative cell viability in the T24 and RT-112 cell lines was measured by MTT assay (mean ± SD, n = 4) after cells were treated for 48 h either with cisplatin, romidepsin or givinostat combined with AZD7762. b Western blot analysis of S345 CHK1 after treatments with gemcitabine (10 nM), cisplatin (5 μM), romidepsin (4 nM) and givinostat (0.5 μM) in T24 cells (24 and 48 h). As loading control, GAPDH was stained. (TIF 214 kb) [file 13046_2016_473_MOESM2_ESM.tif]

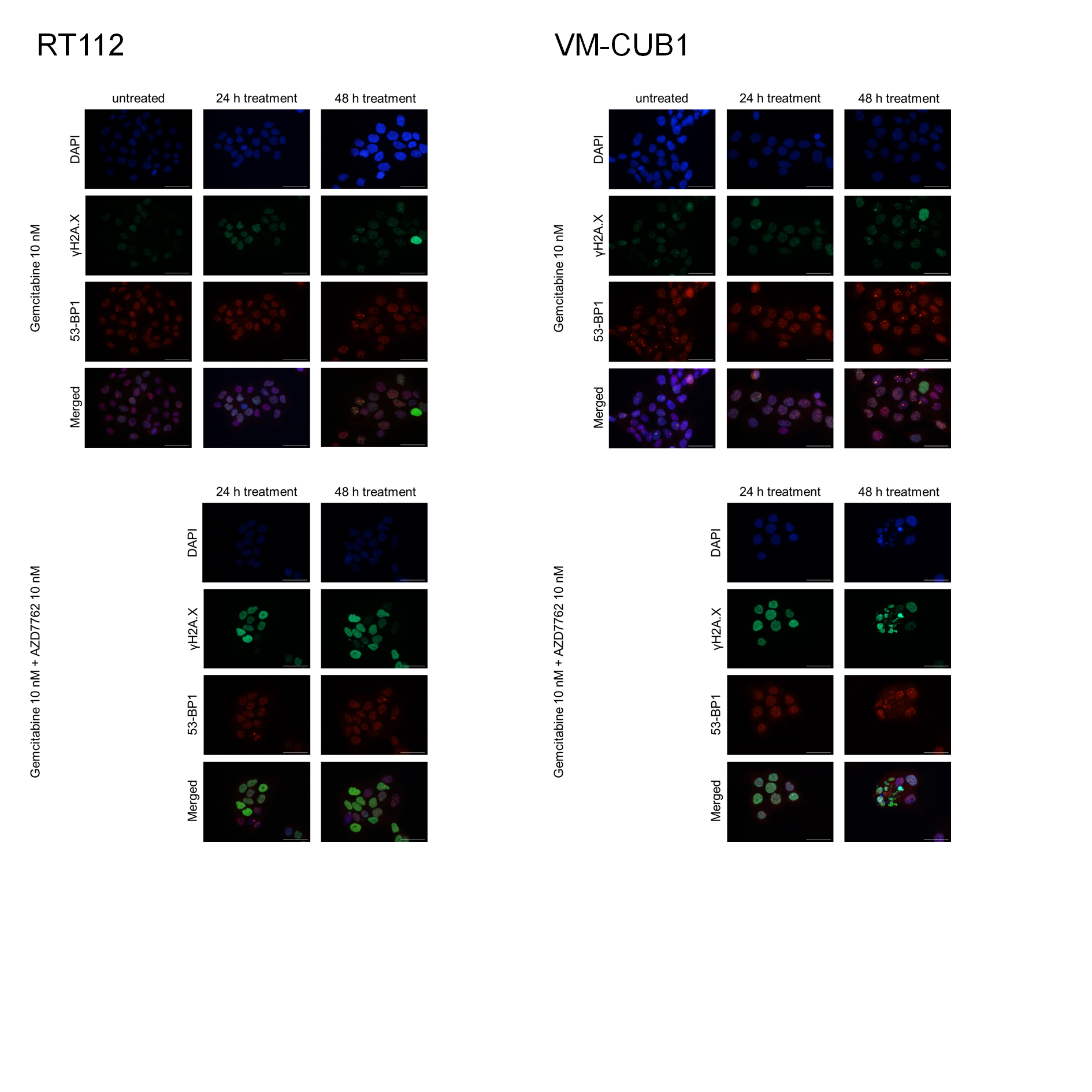

Supplement: Additional file 3: Figure S3. — a, b Immunofluorescence staining of γH2A.X (green), 53-BP1 (red), and nuclei staining with DAPI (blue) in VM-CUB1 (a) and RT-112 (b) cells after the indicated treatments. Scale bar = 50 μm. (TIF 1728 kb) [file 13046_2016_473_MOESM3_ESM.tif]

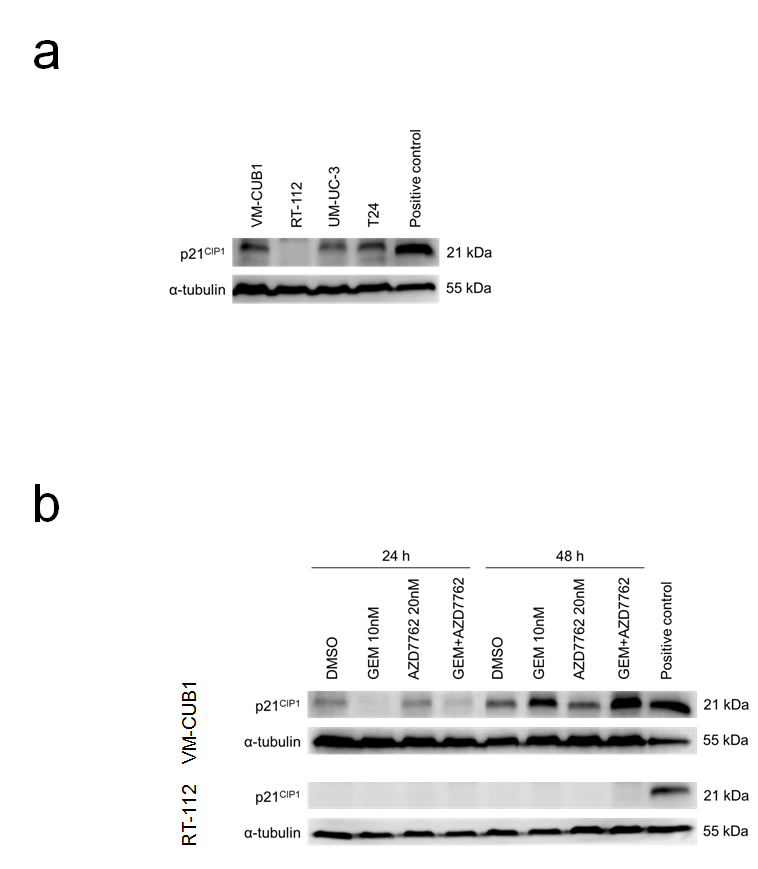

Supplement: Additional file 4: Figure S4. — a CDKN1A mutations in UCCs. p21CIP1 expression of UCCs was assessed by western blotting with α-tubulin as loading control. b Induction of p21CIP1 was assessed by western blot analysis after 24 or 48 h of treatment with gemcitabine and/or AZD7762 in CDKN1A-wildtype VM-CUB1 and CDKN1A-mutant RT-112 cells. (TIF 126 kb) [file 13046_2016_473_MOESM4_ESM.tif]
